# Intergenerational transmission of parental neuroticism to emotional problems in 8‐year‐old children: Genetic and environmental influences

**DOI:** 10.1002/jcv2.12054

**Published:** 2021-11-30

**Authors:** Helga Ask, Espen M. Eilertsen, Line C. Gjerde, Laurie J. Hannigan, Kristin Gustavson, Alexandra Havdahl, Rosa Cheesman, Tom A. McAdams, John M. Hettema, Ted Reichborn‐Kjennerud, Fartein A. Torvik, Eivind Ystrom

**Affiliations:** ^1^ Department of Mental Disorders Norwegian Institute of Public Health Oslo Norway; ^2^ PROMENTA Research Center University of Oslo Oslo Norway; ^3^ Nic Waals Institute Lovisenberg Diaconal Hospital Oslo Norway; ^4^ MRC Integrative Epidemiology Unit University of Bristol Bristol UK; ^5^ Social Genetic & Developmental Psychiatry Centre King's College London London UK; ^6^ Texas A&M Health Science Center Bryan Texas USA; ^7^ Institute of Clinical Medicine University of Oslo Oslo Norway; ^8^ Centre for Fertility and Health Norwegian Institute of Public Health Oslo Norway; ^9^ Department of Psychology University of Oslo Oslo Norway; ^10^ School of Pharmacy University of Oslo Oslo Norway

**Keywords:** anxiety, children‐of‐twins, depression, MoBa, neuroticism

## Abstract

**Background:**

Children of parents with high levels of neuroticism tend to have high neuroticism themselves as well as increased risk of experiencing symptoms of anxiety and depression. It is not yet clear how much of this link is attributable to a potential effect of parent on child (e.g., via a socializing effect) versus to shared genetic risk. We aimed to determine whether there is an intergenerational association after accounting for genetic transmission and assortative mating.

**Methods:**

We used data from the Norwegian Mother, Father and Child Cohort Study including 11,088 sibling pairs in the parental generation, their partners (*N* = 22,176) and their offspring (*N* = 26,091). Exposures were maternal and paternal neuroticism (self‐reported), and the outcomes were neuroticism, symptoms of depression, and symptoms of anxiety in 8‐year‐old children (mother‐reported).

**Results:**

After accounting for assortative mating in parents (phenotypic *r* = 0.26) and genetic transmission (explaining 0%–18% of the mother‐offspring correlations), potential maternal effects explained 80% (95% CI = 47–95) of the association with offspring neuroticism (mother‐child *r* = 0.31), 78% (95% CI = 66–89) of the association with offspring depressive symptoms (*r* = 0.31), and 98% (95% CI = 45–112) of the association with offspring anxiety symptoms (*r* = 0.16). Intergenerational transmission of genetic variants associated with paternal neuroticism accounted for ∼40% (CI = 22%–58%) of the father‐offspring correlations with neuroticism and symptoms of depression (*r* = 0.13 and 0.13, respectively) but none with offspring symptoms of anxiety (*r* = 0.05). The remaining father‐offspring correlations were explained by maternal influences through assortative mating.

**Conclusions:**

These results are consistent with direct effects between maternal and offspring neuroticism and between maternal neuroticism and offspring symptoms of anxiety and depression. Further understanding of these intergenerational processes will require an adequate model of how these constructs (neuroticism, anxiety and depression) relate to each other within generations.

## Graphical abstract

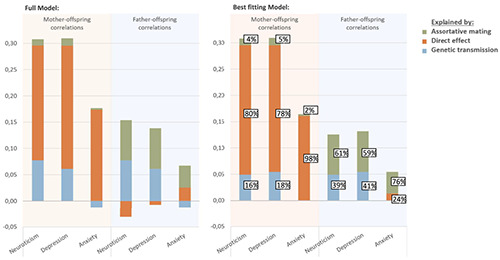

**Key points**

- Children of parents with a stable high level of neuroticism have increased risk of high neuroticism themselves, and of experiencing emotional problems. It is not clear how much of this link is attributable to a potential effect of parent on child versus due to parents and children being genetically similar.
- Our analyses was designed to further our understanding of such intergenerational transmission. Genetic influences (and assortative mating) primarily accounted for father‐child similarities, but mother‐child similarities remained relatively unattenuated.
- Our results indicate that a link between maternal neuroticism and offspring emotional problems remains when genetic transmission is accounted for, suggesting that interventions targeted at maternal functioning could benefit the children.

## INTRODUCTION

Childhood is a risk phase for the development of symptoms and disorders of anxiety (Costello et al., 2003), ranging from transient mild symptoms to full‐blown anxiety disorders. Depressive symptoms and disorders are also prevalent in children and adolescents (Bernaras et al., 2019). Several studies indicate that parents play a role in the development of emotional problems in children—by transmitting genes associated with risk or resilience, and by providing specific parenting styles and rearing environment to their offspring (Ask et al., 2021; Cheesman et al., 2020; Jami et al., 2021). One possible pathway—for both genetic and environmental influences—could work through parental neuroticism.

Neuroticism is a personality trait characterized by emotional instability, stress vulnerability, the tendency to experience intense negative emotions and cognitions, and impulsive behaviors under emotional strain (Pervin & John, 1999). Parents' neuroticism has consistently been linked with negative parental practices (e.g., low interpersonal warmth, inconsistency in strictness, and negative beliefs in ability to regulate the child) (Prinzie et al., 2009; Ystrom et al., 2012) and low spousal relationship satisfaction (Botwin et al., 1997), that in turn could be linked with emotional problems in children. Other negative outcomes observed among offspring of parents high on neuroticism, include poor social functioning, ineffective coping skills, lower educational attainment, and behavioral problems (Ellenbogen et al., 2010).

The processes linking parental neuroticism to child emotional problems are not fully understood. For example, we do not know to what extent the parent‐offspring associations can be explained by *genetic transmission*. Interindividual variation in neuroticism is influenced by genetic factors, with an average heritability estimate of 37% in adulthood (Vukasović & Bratko, 2015) and low to moderate heritability (0%–43%) in childhood (De Fruyt et al., 2006). Substantial genetic correlations between neuroticism and symptoms and disorders of anxiety and depression (Hettema et al., 2006; Nagel et al., 2018) have also been reported, suggesting that genetic influences could explain not only associations with offspring neuroticism but also with offspring symptoms of anxiety and depression.

Parent–offspring associations could also be due to *direct effects* of parental traits on offspring outcomes (McAdams et al., 2014). Direct effects from parental neuroticism to offspring emotional problems could be explained by several different mechanisms including negative parental influences, marital instability, disorganized home environments, or by children learning by or modelling the parental behavior. Maternal neuroticism could also influence behavior and lifestyle during pregnancy as prenatal risk exposures for later emotional problems in children.

A meta‐study concluded that neuroticism in adults was not the result of direct effects of parental neuroticism, and that parent‐offspring resemblance was solely due genetic transmission (Lake et al., 2000). However, these results appear not to generalize to the child and adolescent population. Eley and colleagues found that the association between parent neuroticism and adolescence symptoms of anxiety was not attributable to genetic transmission (Eley et al., 2015). Such a pattern of results is consistent with exposure to parental neuroticism playing a role in the development of neuroticism and symptoms of anxiety in offspring. Direct effects have also been observed in studies on children's depressive symptoms (McAdams et al., 2014). However, these studies were likely inadequately powered to detect modest genetic transmission effects (e.g., Silberg et al., 2010; Singh et al., 2011) and often did not include paternal measures.

Individuals with mental disorders tend to have partners who also have mental disorders (Nordsletten et al., 2016). Assortative mating in heritable traits may influence the genetic and environmental resemblance between family members (Rijsdijk & Sham, 2002) and bias any etiologic decomposition of the intergenerational association if not accounted for. To fully understand the intergenerational transmission of neuroticism, it is important to include estimates of assortative mating. Intergenerational study designs that allow for jointly estimating heritability and assortative mating can also clarify the extent to which non‐random mating in neuroticism could impact the heritability, comorbidity, and prevalence of common mental disorders (Peyrot et al., 2016).

We aim to use an extended version of the children‐of‐twin design to estimate to what extent there is a direct effect of parental neuroticism on offspring neuroticism, symptoms of anxiety, and symptoms of depression after accounting for genetic transmission *and* assortative mating. We use genetically informative data from extended families participating in the Norwegian Mother, Father and Child cohort study (MoBa) comprising data on adult siblings, their spouses and their children assessed at 8 years of age.

The concepts of neuroticism and symptoms of anxiety and depression are closely related (Jylhä & Isometsä, 2006). We did not set out this study to increase our understanding of the structural relationship between them (i.e., as components or pre‐cursors of each other). However, our data provide us with a snapshot of their co‐presence in 8‐year‐old children, and with a unique possibility to investigate the importance of parental neuroticism.

## MATERIALS AND METHODS

### Study sample

**FIGURE 1:**
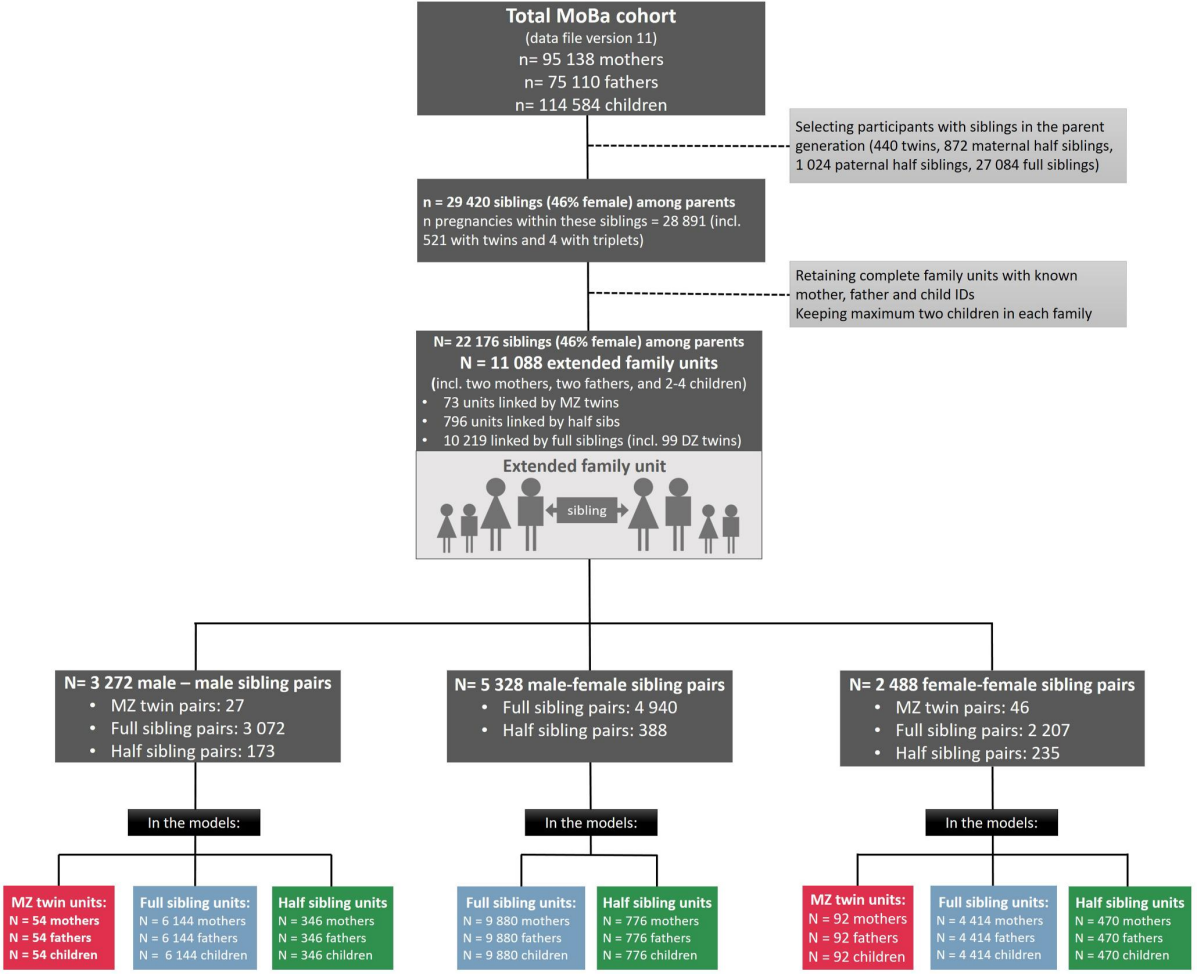
Flow chart describing our sample. The number of children included in our models (lower boxes) assume every family participated with two children each. Families participating with only one child were treated as with missing data on the second child. We used full information maximum likelihood to account for missing in our models. MZ = monozygotic, DZ = dizygotic twins

MoBa is a prospective population‐based pregnancy cohort study conducted by the Norwegian Institute of Public Health (Magnus et al., 2016). Pregnant women were recruited from all over Norway from 1999 to 2008. The women consented to participation in 41% of the pregnancies (see Nilsen et al., 2009 for a description of sample representativeness). The total cohort includes 114,500 children, 95,200 mothers, and 75,200 fathers. The current study is based on version 11 of the quality‐assured data files released for research in 2018. MoBa data have been linked to Norwegian registry data containing pedigree information, and the zygosity of the participating twins has been registered (for details, see Gjerde et al., 2019). For this paper we used sibling pairs from the parental generation, their partners and children. We included a maximum of two offspring per family unit. Our final sample consisted of 11,088 extended family units. There was a total of 26,091 children (48.9% girls) within these families including 4225 sibling pairs. Figure 1 shows the sample selection and final numbers included in our models. Our outcome measures were based on questionnaire data collected when the children were 8 years old. Of the extended families in our sample, 70% returned ratings on the outcome measures for at least one child.

The establishment of MoBa and initial data collection was authorized under a license from the Norwegian Data protection agency and approval from The Regional Committees for Medical and Health Research Ethics. The MoBa cohort is based on regulations from the Norwegian Health Registry Act. The current study was approved by The Regional Committees for Medical and Health Research Ethics.

### Measures

**Parental neuroticism** was defined by a latent variable composed of two questionnaire measures: (1) the sum score on 10 items from the International Personality Item Pool (IPIP) Big‐Five factor markers (Goldberg, 2001). Statements were rated on a five‐point scale (strongly disagree to strongly agree). Cronbach's alpha reliability was 0.89 in mothers and 0.84 in fathers; (2) a composite score of eight symptoms of anxiety and depression from the Hopkins Symptom Checklist (Strand et al., 2003), three anger items from the differential emotions subscale (Izard et al., 1993), and four items from the Rosenberg self‐esteem scale (Blascovich & Tomaka, 1991), previously shown to provide a valid measure of neuroticism (Ystrom et al., 2009). Cronbach's alpha reliability was 0.85 among mothers and 0.79 among fathers. In supporting information S1, more details on the definition (Methods S1, Figure S1), dimensionality (Results S1, Figures S2 and S3), and measurement invariance (Tables S1–S3) of parental neuroticism is provided. Sensitivity analyses were performed including only the IPIP‐items as a measure of neuroticism.

**Offspring neuroticism** was measured with the Hierarchical Personality Inventory for Children (Mervielde & De Fruyt, 1999). We used the sum of six items from the Norwegian short form (NHiPIC‐30) (Vollrath et al., 2016), each referring to a specific behavior. Items were rated by mothers on a five‐point Likert scale (not typical to very typical). Cronbach's alpha reliability was 0.78.

**Offspring symptoms of depression** were measured with the sum of 13 items from the Short Mood and Feelings Questionnaire (Ancold & Stephen, 1995). The items reflect how the child has felt/behaved during the two last weeks and were rated by mothers on three response options (not true, sometimes true, and true). Cronbach's alpha reliability was 0.79.

**Offspring symptoms of anxiety** were measured using the sum of five items from the Screen for Child Anxiety Related Disorders (SCARED) (Birmaher et al., 1999) designed to screen for different anxiety disorders. Mothers rated statements describing how their child has felt/behaved recently using a three‐point Likert scale (not true, sometimes true, and true). Cronbach's alpha was 0.47.

### Covariates

Parity and offspring sex were included as covariates.

### Analyses

The use of samples of families consisting of twins and siblings with children to examine intergenerational associations has been discussed in detail previously (D'onofrio et al., 2003; McAdams et al., 2018). The design compares the similarity between individuals sharing different degrees of genetic relatedness to partition intergenerational associations into environmental and genetic components (McAdams et al., 2018). Within the parental generation of MoBa there are monozygotic twins (sharing all their genes), dizygotic twins (sharing 50% of segregating genes), full siblings (sharing 50% of segregating genes), and half siblings (sharing 25% of segregating genes). Children of monozygotic twins are genetically alike half‐siblings, whereas children of dizygotic twins are like ordinary cousins. This allows us to separate genetic and environmental variance, and provides a counterfactual condition that can be used to answer the question: What would happen if two individuals inherited the same genes, but grew up in different families? If a child is more similar to her mother than to her aunt, who is genetically identical to her mother, that indicates effects operating through the environment (and similarly for father and uncles). On the other hand, if the similarity between relatives is proportional to their genetic relatedness, that indicates genetic transmission. Inclusion of siblings and half‐siblings in the parent generation increases the power of the design, compared to only investigating twins. Including multiple children per family also increases the statistical power (McAdams et al., 2018).

**FIGURE 2:**
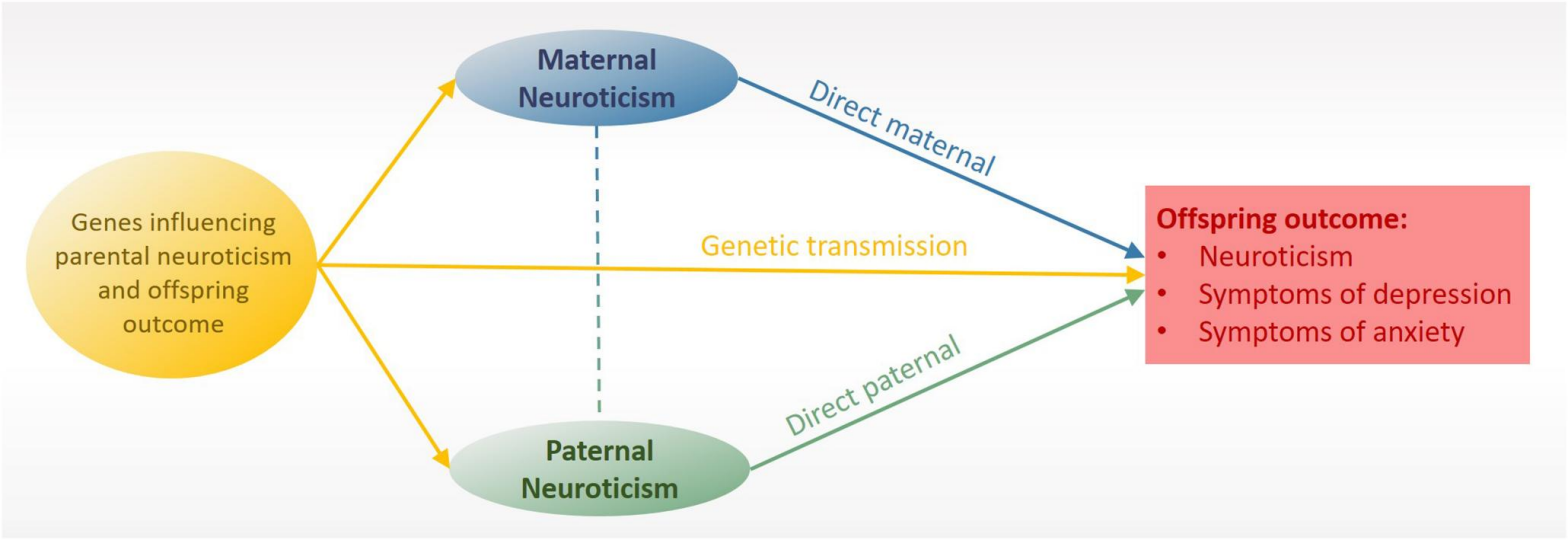
Illustration of our model (see Supporting information Figure S2 for a detailed illustration of the biometric model used). The main goal of our analyses was to estimate the associations between parental neuroticism and offspring outcome after adjusting for genetic transmission. The dashed line between maternal and paternal neuroticism indicates that the model accounts for level of assortative mating (d). Circles indicate latent factors (see Supporting information Figure S1 for a description of the parental neuroticism factor)

Figure 2 presents the main elements of the model. The direct maternal and paternal pathway captures any association between parent and child phenotype not attributable to intergenerational genetic transmission. In our models we assumed that the genetic influences were equal for mothers and fathers, but we estimated the direct transmission independently for mothers and fathers. Assortative mating for neuroticism can bias the results in the direction of environmental transmission, if not accounted for. The inclusion of both mothers and fathers in our study allows us to estimate and account for assortative mating (d) in the intergenerational estimates (Torvik et al., 2020).

As a first step we investigated to what extent there were shared environmental influences on parental neuroticism. If shared environment in the parent generation was estimated to be zero, it would not be necessary to include it in our full model. Previous research on adult neuroticism (Lake et al., 2000) indicates that shared environmental influences are small or non‐existent.

For each offspring outcome (neuroticism, anxiety, depression), the fit of four models, nested within the full model, with constraints to test different assumptions for the transmission were compared: (1) assuming no maternal environmental transmission (Model 2a); (2) assuming no paternal environmental transmission (Model 2b); (3) assuming no environmental transmission (Model 2c); and (4) assuming no genetic transmission (Model 3). We used likelihood ratio tests and Akaike Information Criterion (AIC) value to compare the model fit. A low value of AIC is preferred.

The analyses were conducted in R (Venables et al., 2009) version 3.4.4 using the structural equation modeling package OpenMx (Neale et al., 2016) version 2.11.5. We fitted models to the data using full information maximum likelihood estimation, to account for missing data. A detailed description of the modeling approach is provided in Methods S2 and Figure S4 in supporting information S1.

## RESULTS

Descriptive statistics for the study variables are included in Table S4. Intergenerational correlations are presented in Table 1. Father‐child correlations were generally lower than mother–child correlations. Correlations between parental neuroticism and child anxiety symptoms were lower than those between parental neuroticism and child neuroticism and depressive symptoms. Offspring neuroticism correlated 0.36 (95% CI = 0.35–0.37) with anxiety symptoms and 0.50 (95% CI = 0.49, 0.5) with depressive symptoms. The correlation between symptoms of anxiety and depression was 0.24 (95% CI = 0.23, 0.25).

**TABLE 1:** Raw Intergenerational correlations (with 95% CI in brackets)

Neuroticism in…	Child neuroticism	Child symptoms of depression	Child symptoms of anxiety
…mother	0.23 (0.20, 0.25)	0.23 (0.21, 0.26)	0.13 (0.10, 0.16)
…father	0.09 (0.06, 0.12)	0.11 (0.08, 0.14)	0.04 (0.01, 0.07)
…aunt	0.03 (0.00, 0.06)	0.05 (0.02, 0.08)	0.01 (−0.02, 0.04)
…uncle	0.03 (0.00, 0.06)	0.03 (0.00, 0.06)	0.02 (−0.01, 0.05)

*Note*: Neuroticism = the sum of the two neuroticism measures in parents. Correlations were based on outcome measures from the first child in one of the extended family units linked by parental siblings.

The parental C effect was estimated to be zero; therefore, we did not include C or intergenerational C influences in our main models. Table 2 presents the results of the model fitting. For all outcomes, the model assuming no direct effects (Model 2c) provided the worst fit to our data according to AIC (i.e., highest AIC value). The model assuming no paternal transmission (Model 2b) had the lowest AIC value for offspring neuroticism and symptoms of depression. Both the paternal effect and the genetic transmission were estimated close to zero in the full model for offspring anxiety symptoms, and the fit of Model 2b and Model 3 was not distinguishable (AIC difference of 0.31). The best fitting models with respect to AIC did not, however, provide a significantly better fit than the full model. Therefore, parameter estimates from both the full and the best fitting models are presented in Table 3. Selection of models does not influence the main conclusions we can draw from our results.

**TABLE 2:** Results of the model fitting for the intergenerational transmission of parental neuroticism to offspring neuroticism, symptoms of depression, and symptoms of anxiety

8‐Year outcome	Model		Comparison	Δ‐2LL	Δ DF	AIC	Δ‐AIC	*p*
***NEUROTICISM***	1	Full model				45,841.70		
	2a	No direct maternal transmission (*p*_*m*_ = 0)	1	39.49	1	45,879.19	37.49	<.001
	**2b**	**No direct paternal transmission (*p***_***f***_ ** = 0)**	**1**	**0.93**	**1**	**45,840.40**	**1.30**	**.401**
	2c	No direct transmission (*p*_*m*_ = *p*_*f*_ = 0)	1	175.88	2	46,013.58	171.88	<.001
	3	No genetic transmission (*g* = 0)	1	4.86	1	45,844.56	2.86	.027
***DEPRESSION***	1	Full model				45,972.32		
	2a	No direct maternal transmission (*p*_*m*_ = 0)	1	45.10	1	46,015.42	43.1	<.001
	**2b**	**No direct paternal transmission (*p***_***f***_ ** = 0)**	**1**	**0.04**	**1**	**45,970.36**	**1.96**	**.831**
	2c	No direct transmission (*p*_*m*_ = *p*_*f*_ = 0)	1	166.66	2	46,134.98	162.66	<.001
	3	No genetic transmission (*g* = 0)	1	3.37	1	45,973.69	1.37	.066
***ANXIETY***	1b	Full model				45,923.26		
	2a	No direct maternal transmission (*p*_*m*_ = 0)	1	18.92	1	45,940.18	16.92	<.001
	2b	No direct paternal transmission (*p*_*f*_ = 0)	1	0.42	1	45,921.68	1.58	.516
	2c	No direct transmission (*p*_*m*_ = *p*_*f*_ = 0)	1	63.49	2	45,982.75	59.49	<.001
	**3**	**No genetic transmission (*g* = 0)**	**1**	**0.11**	**1**	**45,921.37**	**1.89**	**.738**

*Note*: Best fitting models are marked in bold.

Abbreviations: ‐2LL, minus twice the log likelihood; AIC, Akaike Information Criterion.

**TABLE 3:** Unstandardized parameter estimates (with 95% CI in brackets) from the full model and the best fitting model of intergenerational transmission across childhood outcomes

Parameters		Neuroticism		Symptoms of depression		Symptoms of anxiety	
		Full model (1)	Model 2b	Full model (1)	Model 2b	Full model (1)	Model 3
*p*_*m*_	Direct maternal	0.32 (0.21, 0.43)	0.36 (0.31, 0.42)	0.34 (0.24, 0.45)	0.36 (0.30, 0.41)	0.25 (0.15, 0.36)	0.23 (0.20, 0.27)
*p*_*f*_	Direct paternal	−0.05 (−0.15, 0.06)	–	−0.02 (−0.12, 0.09)	–	0.04 (−0.07, 0.14)	0.02 (−0.01, 0.05)
*g*	Genetic transmission	0.25 (0.03, 0.46)	0.16 (0.09, 0.23)	0.21 (0.00, 0.41)	0.18 (0.11, 0.25)	−0.04 (−0.25, 0.17)	–

*Note*: Model 2b: assuming no parental direct effect, Model 3: assuming no genetic transmission.

**FIGURE 3:**
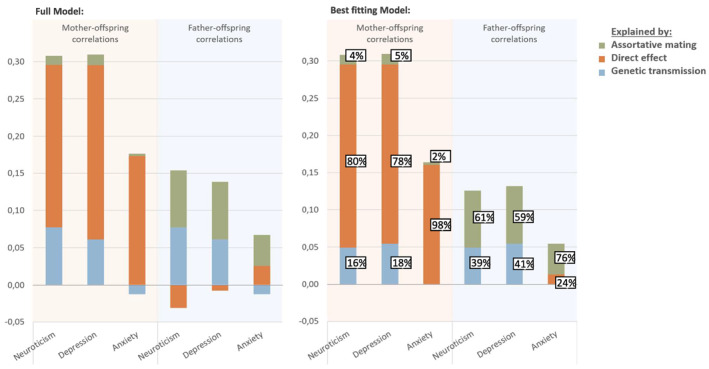
The bars show the size of the correlations between maternal/paternal neuroticism and pre‐pubertal children's scores on neuroticism, symptoms of depression and symptoms of anxiety. Percentage of parent‐offspring correlations explained by direct maternal effects are shown in orange, genetic transmission effects in blue, and due to assortative mating in green. Results based on the full models (right) and the best fitting models (left)

The proportion of the estimated parent‐child correlations explained by genetic transmission, direct influences and assortative mating are shown in Figure 3. In the best fitting model, genetic transmission of maternal neuroticism accounted for 16% (95% CI = 9%–23%) of the correlation with offspring neuroticism (*r* = 0.31, 95% CI = 0.29, 0.33), 18% (95% CI = 11%–25%) of the correlation with offspring depressive symptoms (*r* = 0.31, 95% CI = 0.29, 0.33), and none of the correlation with offspring anxiety symptoms (*r* = 0.16, 95% CI = 0.14, 0.19). Genetic transmission of paternal neuroticism accounted for 39% (95% CI = 22%–56%) of the association with offspring neuroticism (*r* = 0.13, 95% CI = 0.11, 0.15), 41% (95% CI = 25%–58%) of the association with offspring symptoms of depression (*r* = 0.13, 95% CI = 0.11, 0.15), and none of the association with offspring symptoms of anxiety (*r* = 0.05, 95% CI = 0.03, 0.08).

The partner correlation in our model was 0.26 (95% CI = 0.24–0.28). Of this, 61.5% was due to genetic correlation (*r*_*g*_ = 0.16), and the rest to environmental correlation (*r*_*e*_ = 0.10). Due to the assortative mating, and maternal influences (genetic and direct) on offspring outcomes, part of the intergenerational father–offspring similarity was explained through this path (see Figure 3). Pathways through assortative mating and maternal influences explained 61% (95% CI = 54%–67%) of the father‐offspring association on neuroticism, 59% (95% CI = 52%–65%) of the association with symptoms of depression and 76% (95% CI = 11%–89%) of the association with symptoms of anxiety. Pathways through assortative mating and paternal influences explained <5% of the mother‐offspring similarity. In mothers, the direct path explained 80% (neuroticism, 95% CI = 47%–95%), 78% (depression, 95% CI = 66%–89%), and 98% (anxiety 95% CI = 45%–112%) of the mother–child similarity.

In total, intergenerational influences (direct and genetic) explained 8.5%, 8.8%, and 2.7% of the variance in childhood neuroticism, symptoms of depression and symptoms of anxiety, respectively. Figure S5 shows the proportion of outcome variance explained by transmission and child‐specific influences. In models assuming no influences of common environment in childhood (see Results S2 and Tables S5–S8 in supporting information S1 for more details) genetic influences specific to childhood neuroticism, symptoms of depression, and symptoms of anxiety explained 23.9%, 40.8%, and 27.8% of the variance in children, respectively. The total heritability for the offspring variables was 26.3% for neuroticism, 43.7% for depressive symptoms, and 27.8% for anxiety symptoms. Sensitivity analyses including only items from N1 (the IPIP measure of neuroticism) were run. These results, and their interpretation, are included in Results S2 and Tables S9–S12 in supporting information S1.

Our models estimated the heritability of neuroticism in the adult generation to be 43% (CI: 0.41–0.46). Assortative mating has consequences for the additive genetic variance in future generations (Lynch & Walsh, 1998). Knowing the genetic correlation between spouses (*r*_*g*_ = 0.16), we can estimate what the heritability in offspring phenotypes would be without generations of assortative mating (from a random mating base population to asymptotically approaching equilibrium (Lynch & Walsh, 1998)**.** Due to assortative mating the heritability of neuroticism, depression, and anxiety in the offspring generation increased by, respectively 18%, 19%, and 19% compared to a hypothetical situation without such mating.

Failing to account for the assortative mating in intergenerational models could also influence the intergenerational estimates. We ran our models fixing the level of assortative mating to be zero. Despite having an additional degree of freedom from this constraint, the model had worse fit to our data (Table S13), and the results indicated slightly elevated estimates of genetic transmission (Table S14).

## DISCUSSION

We used an extended children‐of‐twins design to investigate the modes of intergenerational transmission of parental neuroticism to neuroticism and symptoms of depression and anxiety in children. Our results are consistent with genetic transmission and a direct effect of maternal neuroticism on offspring outcomes.

### Maternal influences

After correcting for genetic influences, the direct maternal path explained 78%–98% of the mother‐child similarity. These maternal influences are in line with previous children‐of‐twins studies on offspring neuroticism (Eley et al., 2015), anxiety (Ahmadzadeh et al., 2019; Eley et al., 2015) and depression (McAdams et al., 2015; Silberg et al., 2010; Singh et al., 2011) and a recent molecular genetic study (Jami et al., 2020). Another molecular genetic study using MoBa data also suggest that parental genes have an indirect (environmentally mediated) effect on offspring symptoms of depression, but not on anxiety (Cheesman et al., 2020). Several possible mechanisms can explain the direct transmission between mothers and children. For example, a maternal stable high level of neuroticism might lead to inconsistent parenting and a disorganized home environment for their children. This personality trait has also been associated with marital instability, a factor previously associated with negative outcomes in children (Prinzie et al., 2009). The association could also reflect a process of children learning from or modelling the mother's behavior or reaction patterns. Importantly, although we modelled effects of mothers on children, it is conversely possible that the behavior of a child with high neuroticism or symptoms of anxiety and depression increases maternal neuroticism. In addition, environmental factors that parents and children share, such as neighborhood, might act as “third variables” that could influence both the stable level of neuroticism in mothers and neuroticism, and symptoms of anxiety and depression in 8‐year‐old children. Future studies should further investigate such potential mechanisms. Also, a developmental perspective should be included since the maternal effects may not persist until adulthood, as a large meta‐study found no direct transmission in neuroticism measured in adulthood (Lake et al., 2000).

Our results reflect processes associated with stable maternal neurotic traits and do not provide knowledge on how maternal neuroticism at different time points during child development might have differential effects. Timing of measurement could influence the results. For example, previous studies have found maternal depression after pregnancy, but not during pregnancy to influence children's emotional problems (Gjerde et al., 2021; Hannigan et al., 2018)

### Father‐child similarity

The direct paternal path explained 0% of father‐child correlations for child neuroticism and symptoms of depression, and 24% of the correlation between paternal neuroticism and offspring symptoms of anxiety. This father–child correlation (stable parental neuroticism with offspring anxiety) was, however, very low (*r* = 0.05). Most of the father–child similarity (59%–76%) was explained by assortative mating, that the fathers resemble their children because they resemble the mother. The partner correlation in neuroticism was moderate (0.26).

### Genetic transmission

Our results indicate that child neuroticism, anxiety and depressive symptoms are modest‐to‐moderately genetically influenced. However, most of this genetic influence is not shared with the genetic influences on the stable neuroticism trait in adults. In our models, genetic transmission explained 16% and 18% of the associations between mothers' stable neuroticism and children's neuroticism and depressive symptoms, respectively. This corresponded to 39% and 41% of the relatively lower father‐child correlations. Unlike depression and neuroticism, there were no evidence of genetic overlap between parental neuroticism and symptoms of anxiety in offspring. This finding is in line with previous studies on parental anxiety and parenting behavior and internalizing symptoms in offspring (Jami et al., 2021). The high level of child‐specific genetic factors may indicate that different genetic liability underlies the traits measured in childhood and adulthood. This appears to contrast with conclusions from the literature suggesting genetic influences as a driving factor for stability in symptoms of anxiety and depression (Hannigan et al., 2017). Several lines of research support developmental dynamic effects, particularly the findings that the genetic factors change across the life span for emotional problems (Gillespie et al., 2004; Nivard et al., 2015) and that neuroticism is less heritable among children than among adults (Rice et al., 2002). Such findings are corroborated by recent findings from molecular genetics. For example, genetic association studies have found adult major depressive disorder risk alleles (polygenic risk scores) to be robustly associated with emotional problems in adulthood (Okbay et al., 2016) but show little evidence of association with emotional problems in children (Jansen et al., 2018; Riglin et al., 2018). Interestingly, they suggest that childhood emotional problems may share more genetic risk with schizophrenia and neurodevelopmental disorders (Lee et al., 2019; Rice et al., 2019). On the other hand, a recent meta‐study of birth cohorts found polygenic risk for neuroticism to predict emotional problems in childhood (Akingbuwa et al., 2020). However, when such polygenic risk scores are not separated into transmitted and untransmitted alleles, the observed associations could indicate both direct genetic effects from the child's genetic variants, or indirect genetic effects from the parents' genetic variants mediated through the environment. The possible dynamic genetic architecture from pre‐puberty to adulthood, support the implementation of childhood‐specific genome‐wide association studies on neuroticism and emotional problems.

### Limitations

The current study has several strengths; the use of a large population‐based sample and inclusion of two children per family and both mothers and fathers in the design. In addition, modelling parental neuroticism as a latent factor allowed us to partition out unsystematic and occasion/measure‐specific measurement error. Yet, there are some important limitations. First, the participation rate in MoBa was 41% suggesting the possibility of bias due to nonrandom participation. Using national data from the medical birth registry of Norway, a previous study found that younger women, smokers, and women with low educational level were less likely to participate in MoBa (Nilsen et al., 2009). In line with other pregnancy cohorts, MoBa participants may have healthier lifestyle and higher socioeconomic position than the general population. However, most bivariate associations were unlikely affected by the low participation rate (Nilsen et al., 2009).

Second, the outcome measures were obtained by maternal reports. Studies report only low‐to‐moderate levels of agreement between parents and children for childhood psychopathology (De Los Reyes & Kazdin, 2005). Also, the maternal ratings of their offspring could reflect their own neuroticism or their response style, that would correlate with their own self‐report. Such shared method variance could explain why the children appear to be more similar to their mothers than to their fathers and would be defined as a maternal direct effect in our models. The different patterns in maternal and paternal results should therefore be interpreted with this limitation in mind. Paternal ratings of offspring phenotypes are of great value, and we encourage future studies to include such measures. The potential maternal rating bias is a limitation, yet it should be noted that the use of a longitudinal measure of stable neuroticism reduces the likelihood of this bias affecting our results. Also, several of our findings are not affected by this potential confounding: the level of assortative mating, and that nearly all of the associations with fathers were explained by common genetic influences and assortative mating. In addition, a recently published study on the same sample (Torvik et al., 2020) reported crude observed correlations between level of parental educational attainment and maternally reported offspring depression, which were of approximately the same magnitude from maternal and paternal educational attainment. Nevertheless, it will be of great value to return to this question with self‐report data from the children, which will be available in the future (for older children).

Third, the SCARED measure of anxiety had low internal consistency. This can be explained by the intention to screen for several different anxiety disorders. Increased measurement error could partly explain why the intergenerational model for anxiety differs from neuroticism and depression.

Fourth, it appeared that our models were not adequately powered to distinguish between child‐specific genetic and shared (familial) environmental influences. Therefore, we should be cautious on interpreting the size of these estimates. It is likely that both genetic factors and family environment (and their correlation) influence the child neuroticism and symptoms of depression. Shared environmental influences in child anxiety symptoms have not been identified consistently in previous studies (Jami et al., 2021; Waszczuk et al., 2014). Importantly, these child‐specific estimates do not influence the intergenerational estimates.

Fifth, in our design the parent‐child associations were adjusted for the confounding effect of additive genetic influences. Obviously, there could be other confounding factors in the environment causing similarity within families, that we have not adjusted for. This is a limitation that is relevant to any children of twin design. Such family effects should be investigated using other study designs.

In this paper, we have taken a pragmatic approach to whether anxiety and depression symptoms should be defined as components of neuroticism (as treated in the parental generation), or as distinct entities (as treated in the offspring generation). Our sensitivity analysis, using only the IPIP measure of neuroticism in the parental generation suggested that our findings were not sensitive to this distinction. However, we acknowledge that interpretation and replicability of our findings will depend on appropriate understanding of the interrelationships among these constructs. This is outside the scope of the current paper, but an important topic for advancing our knowledge on how these constructs aggregates within families.

## CONCLUSION

Our results indicate that a link between maternal neuroticism and offspring neuroticism and emotional problems remains when genetic transmission is accounted for. Further understanding of these intergenerational processes will require an adequate model of how neuroticism, anxiety and depression relate to each other within generations.

## CONFLICT OF INTERESTS

Eivind Ystrom is a Joint Editor of JCPP *Advances*. The remaining authors have declared that they have no competing or potential conflicts of interest. Tom A. McAdams is a member of the JCPP *Advances* Editorial Advisory Board. [Corrections made on 22 June 2022, after first online publication: This Conflict of Interests statement has been updated in this version.]

## AUTHOR CONTRIBUTIONS

Ask contributed to the conception and design of the study, performed the analyses, interpreted results, and drafted the initial manuscript; Eilertsen contributed to the conception and design of the study and had a lead contribution in methodology and software. Torvik and Ystrom had major roles in conceptualizing and designing the study and interpreted results. All authors critically reviewed and edited the manuscript and approved the final manuscript as submitted.

## ETHICS STATEMENT

The establishment of MoBa and initial data collection was authorized under a license from the Norwegian Data protection agency and approval from The Regional Committees for Medical and Health Research Ethics. The MoBa cohort is based on regulations from the Norwegian Health Registry Act. The current study was approved by The Regional Committees for Medical and Health Research Ethics.

## CODE AVAILABILITY STATEMENT

Analysis code for running intergenerational models will be available on GitHub.

## Supporting information

Supporting Information S1

## Data Availability

The consent given by the MoBa participants does not open for storage of data on an individual level in repositories or journals. Researchers who want access to data sets for replication should submit an application to datatilgang@fhi.no. Access to data sets requires approval from The Regional Committee for Medical and Health Research Ethics in Norway and an agreement with MoBa.
