# Supplementary material for: Intergenerational transmission of parental neuroticism to emotional problems in 8‐year‐old children: Genetic and environmental influences
Source: JCPP Adv. 2021 Nov 30;1(4):e12054. doi: 10.1002/jcv2.12054 (PMC10242898; doi:10.1002/jcv2.12054)
Supplement: Supplementary file 1 — Supporting Information S1 [file JCV2-1-e12054-s001.docx]

**Supporting information: Intergenerational transmission of parental neuroticism to emotional problems in 8-year old children: genetic and environmental influences**

Helga Ask, Espen M. Eilertsen, Line C. Gjerde, Laurie Hannigan, Kristin Gustavson, Alexandra Havdahl, Rosa Cheesman, Tom A. McAdams, John M. Hettema, Ted Reichborn-Kjennerud, Fartein A. Torvik, Eivind Ystrom

[1. Methods S1: Definition of parental neuroticism 2](#_Toc88565792)

[2. FigS1. Parental neuroticism 2](#_Toc88565793)

[3. Results S1: Parental neuroticism – dimensionality and measurement invariance 3](#_Toc88565794)

[4. FigS2. Scree Plot Parental neuroticism 3](#_Toc88565795)

[5. FigS3. CFA N1 and N2, and their correlation 3](#_Toc88565796)

[6. TableS1: Fit statistics CFA model for items measured at two different time points 4](#_Toc88565797)

[7. TableS2: Measurement invariance N1 (IPIP) 4](#_Toc88565798)

[8. TableS3: Measurement invariance N2 (anxiety, depression, anger, self-esteem) 4](#_Toc88565799)

[9. MethodsS2: Statistical modeling 5](#_Toc88565800)

[10. FigS4. Illustration of the biometric model including parents and two children 6](#_Toc88565801)

[11. TableS4: Descriptive statistics 7](#_Toc88565802)

[12. ResultsS2 8](#_Toc88565803)

[13. TableS5. 9](file:///H:\06SAMARBEID\Eivind\intergenerasjonell%20angst\sub_JCPP-A_revisjon\REV2\SupplementAsk.docx#_Toc88565804)

[14. TableS6. 9](file:///H:\06SAMARBEID\Eivind\intergenerasjonell%20angst\sub_JCPP-A_revisjon\REV2\SupplementAsk.docx#_Toc88565805)

[15. TableS7. 9](file:///H:\06SAMARBEID\Eivind\intergenerasjonell%20angst\sub_JCPP-A_revisjon\REV2\SupplementAsk.docx#_Toc88565806)

[16. TableS8**.** 10](file:///H:\06SAMARBEID\Eivind\intergenerasjonell%20angst\sub_JCPP-A_revisjon\REV2\SupplementAsk.docx#_Toc88565807)

[17. TableS9 11](#_Toc88565808)

[18. TableS10. 11](file:///H:\06SAMARBEID\Eivind\intergenerasjonell%20angst\sub_JCPP-A_revisjon\REV2\SupplementAsk.docx#_Toc88565809)

[19. TableS11 12](#_Toc88565810)

[20. TableS12. 12](file:///H:\06SAMARBEID\Eivind\intergenerasjonell%20angst\sub_JCPP-A_revisjon\REV2\SupplementAsk.docx#_Toc88565811)

[21. TableS13 13](#_Toc88565812)

[22. TableS14. 13](file:///H:\06SAMARBEID\Eivind\intergenerasjonell%20angst\sub_JCPP-A_revisjon\REV2\SupplementAsk.docx#_Toc88565813)

[23. FigS5. 14](file:///H:\06SAMARBEID\Eivind\intergenerasjonell%20angst\sub_JCPP-A_revisjon\REV2\SupplementAsk.docx#_Toc88565814)

[**References** 15](#_Toc88565815)

# Methods S1: Definition of parental neuroticism

Parental neuroticism was defined by a latent variable composed of two questionnaire measures (N1 and N2, see Box1). N1 was not included in all the versions of the questionnaires and was completed by 23.6% of the mothers and 39.5% of the fathers. N2 was completed by 83.2% of mothers and 88.7% of fathers. The z-scaled mean scores from the three N2 components (Hopkins symptom checklist, Anger subscale, and Rosenberg self-esteem scale) were summed.

N1 was included in the father questionnaire at the 17^th^ week of gestation and in the mother questionnaire when the children were 5 years old. N2 was included in the father questionnaire at the 17^th^ week of gestation, and in the mother questionnaire at the 30^th^ week of gestation. Since our sample included mothers and fathers participating with up to two children in MoBa, each parent was assessed by N1 and N2 at up to two time points. As FigS1 illustrates, the neuroticism factor therefore loaded on up to four scores for each parent. Parents participating with only one child, or on only one of the measures were registered with missing data on these scores.

**N1 =** 10 items from the International Personality Item Pool (IPIP)

**N2 =** 8 items from the Hopkins

Symptom Checklist (SCL-8)

**+** 3 items from the anger subscale of

the Differential Emotions Scale

**+** 4 items from the Rosenberg self-

esteem scale

Treating neuroticism as a latent factor allowed us to use all the available information (e.g. use the data on N1 despite lower participation rate), and to tap the stable trait of neuroticism, as it represents what is common across time points. Using a latent factor also removes unsystematic and occasion/measure-specific forms of measurement error form our model.

Box1

As a sensitivity analyses, all our models were run including only the N1 measure

# FigS1. Parental neuroticism


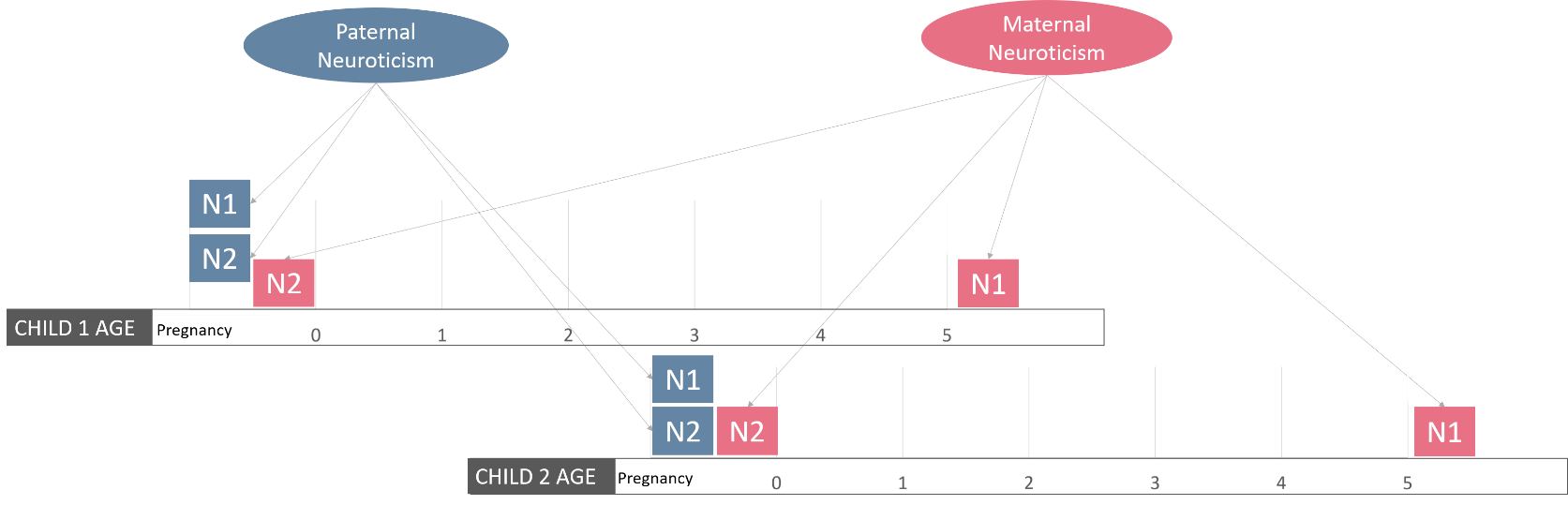


# Results S1: Parental neuroticism – dimensionality and measurement invariance

We performed an exploratory factor analyse (EFA) on the paternal responses (during pregnancy) on the 25 items included in our neuroticism measure. The EFA was performed using categorical variables in Mplus, version 8 (Muthén, 2010). Since several fathers participated more than once the father ID was included as a cluster variable (N observations=77 023, N fathers=67 022). The results indicated good fit for a one-factor solution (RMSEA = 0,05 with 90% CI .049, .050, CFI=0.882, TLI=0.872), with loadings ranging from 0.50-0.94. EFA solutions with more than one factor did, however provide a better fit to our data. This is a usual observation when investigating the dimensionality of neuroticism – that the dependencies of facet items lead to a better fit for models with more than one latent factor. Such issues are usually solved by aggregating into facets before running the factor analysis. The Scree plot in FigS2 shows the dimensionality of the IPIP items, together with mean scores of SCL-8, self-esteem and anger, indicating that these (mean) items can be represented by one dimension together with the IPIP items.

# FigS2. Scree Plot Parental neuroticism


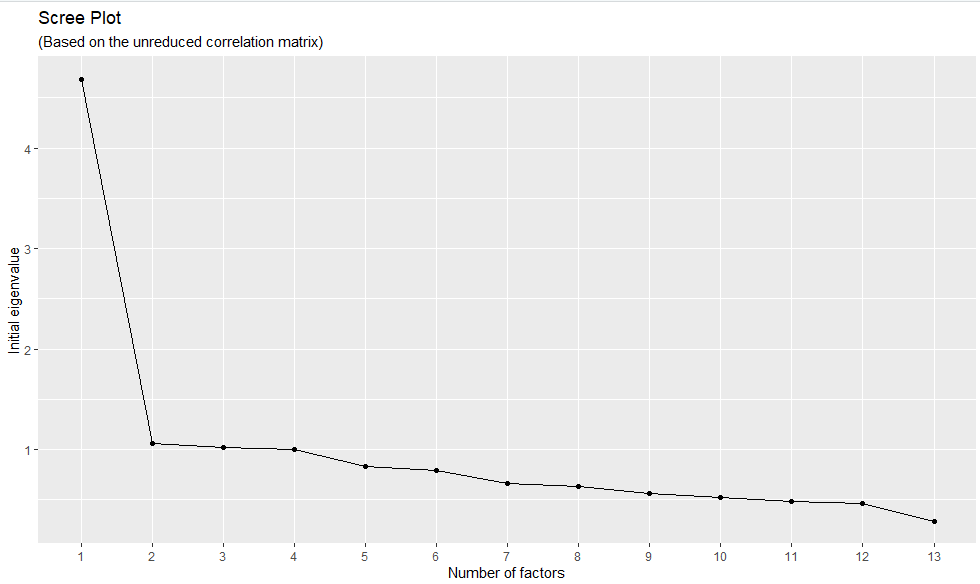


We ran a confirmatory factor analysis (CFA) to investigate to what extent the IPIP measure of neuroticism (N1), with a previously established internal structure and our suggested alternative measure (N2) were capturing the same underlying construct. The figure below (FigS3) shows that the combination of (latent) self-esteem, (latent) anger, and (latent) anxiety/depressive symptoms capture a latent construct correlating 0.92 with the latent N1 measure. These results were also presented in a previous study using MoBa data (Ystrom, Barker, & Vollrath, 2012), suggesting that N2 provides a valid measure of neuroticism.

# FigS3. CFA N1 and N2, and their correlation


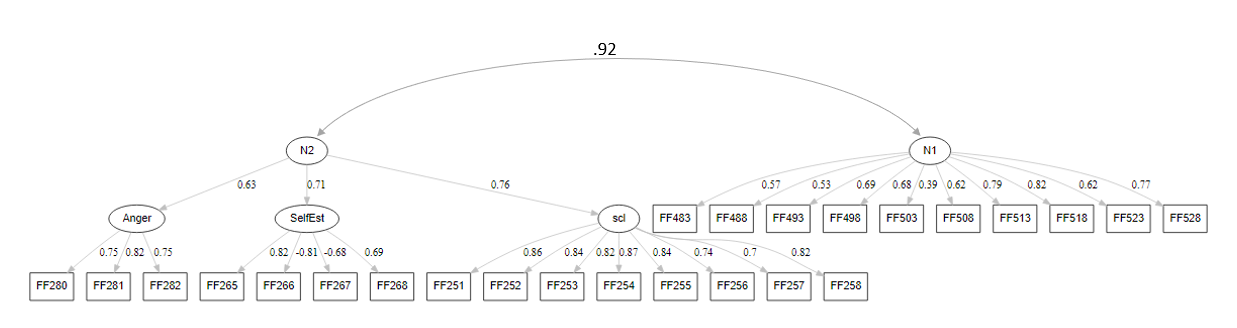


We took some steps to ensure measurement invariance in neuroticism. First, we ran our measurement model (FigS3) using items from time 1 and items for time 2, separately. Fit statistics for the two models are included in TableS1.

# TableS1: Fit statistics CFA model for items measured at two different time points

| Fit statistics | Model_time1  N=30 835 fathers | Model_time2  N=1 130 fathers |
| --- | --- | --- |
| CFI | 0.976 | 0.983 |
| TLI | 0.973 | 0.981 |
| RMSEA | 0.062 | 0.055 |
| SRMR | 0.059 | 0.062 |
| Guidelines for model fit indices: (1) Comparative fit index (CFI) > .95; Tucker-Lewis index (TLI) > .95; and root mean square error of approximation (RMSEA) < .06. With sufficient model fit for configural invariance, we can proceed to metric invariance. | | |

The fit indices show us that the factorial structure holds across both timepoints. To test for measurement invariance, we estimated and compared increasingly constrained CFA models with each other using the semTools package (Jorgensen et al., 2018) in R. We tested each of the two measures (N1 and N2) separately. More restricted models were compared to the previous model using a chi-square difference (Δ χ²) test. If not significant, invariance is established.

**Measurement invariance models:**

Model1 (fit.configural): Configural invariance. The same factor structure is imposed on all units

Model2 (fit.loadings): Weak invariance. The factor loadings are constrained to be equal across units.

Model3 (fit.intercepts): Strong invariance. The factor loadings and intercepts are constrained to be equal

Model4 (fit.residuals): Strict invariance. The factor loadings, intercepts, and residual variances are

constrained to be equal across units

Model5 (fit.means): The factor loadings, intercepts, residual variances and means are

constrained to be equal across units

# TableS2: Measurement invariance N1 (IPIP)


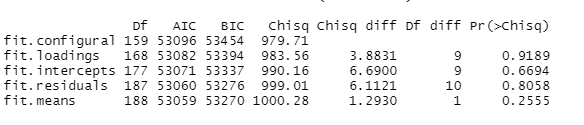


# TableS3: Measurement invariance N2 (anxiety, depression, anger, self-esteem)


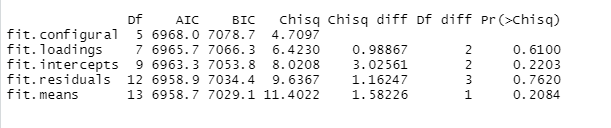


The results for N1 (TableS2) and N2 (TableS3) show that the constrained model fits the data equally well, thus measurement invariance can be assumed.

# MethodsS2: Statistical modeling

Our modeling approach to intergenerational transmission is based on that used by Silberg et al. (2010). The children-of-twins model can be seen as an extension of the classic ACE model for twin designs, where the phenotype is decomposed into an additive genetic component (A), a shared environmental component (C) and a unique environmental component (E) (D'Onofrio et al., 2003; McAdams et al., 2014; Neale & Maes, 2004). In addition to pairs of siblings, we extend this design with the inclusion of partners and children. This allows us to compare alternative models of intergenerational transmission.

The path diagram in FigS2 shows the essential features of the model for a nuclear family. Latent variables are depicted by circles and observed variables by rectangles. For mothers and fathers, the two neuroticism measures from each measurement occasion is treated as a function of a latent neuroticism value. We describe the latent parental neuroticism values as a function of additive genetic effects ($A$) and environmental effects unique to the individual ($E$). The strength of these influences is estimated from the path coefficients $a$ and $e$ depicted by the arrows in the figure. The covariance between maternal and paternal neuroticism is estimated with the parameter $d$. We model similarity in underlying neuroticism between partners as phenotypic assortment, meaning that we assume that partners have selected each other based on their levels of neuroticism. Such assortment would induce correlated genetic and environmental effects between partners. These implications are represented with the horizontal line between maternal and paternal neuroticism values.

The different measures of offspring emotional problems were treated as observed variables. To model joint contributions of genetic effects across generations, we split the additive genetic effect into two components, one shared with parental neuroticism ($A_{1}^{'}$) and one unique to offspring symptoms ($A_{2}$). This allows different genes to be expressed across generations. For example, if the same genes contribute to variability in both generations, the coefficient from $A_{2}$ is expected to be zero. On the other hand, if completely different genes are expressed across generations, the coefficient from $A_{1}^{'}$ is expected to be zero. Direct effects of maternal and paternal neuroticism on offspring emotional problems are depicted with the arrows with coefficients $p_{m}$ and $p_{f}$, respectively. In addition to the effects of parental neuroticism we assume that offspring emotional problems may be influenced by environmental effects common to siblings ($C$) and environmental effects unique to the individual ($E$).

With this model structure, two distinct sources of intergenerational transmission are represented. Children may resemble parents due to inheritance of genes contributing to both parental neuroticism and offspring emotional problems, but they may also resemble parents due to direct effects through the environment.

1. FigS4. Illustration of the biometric model including parents and two children


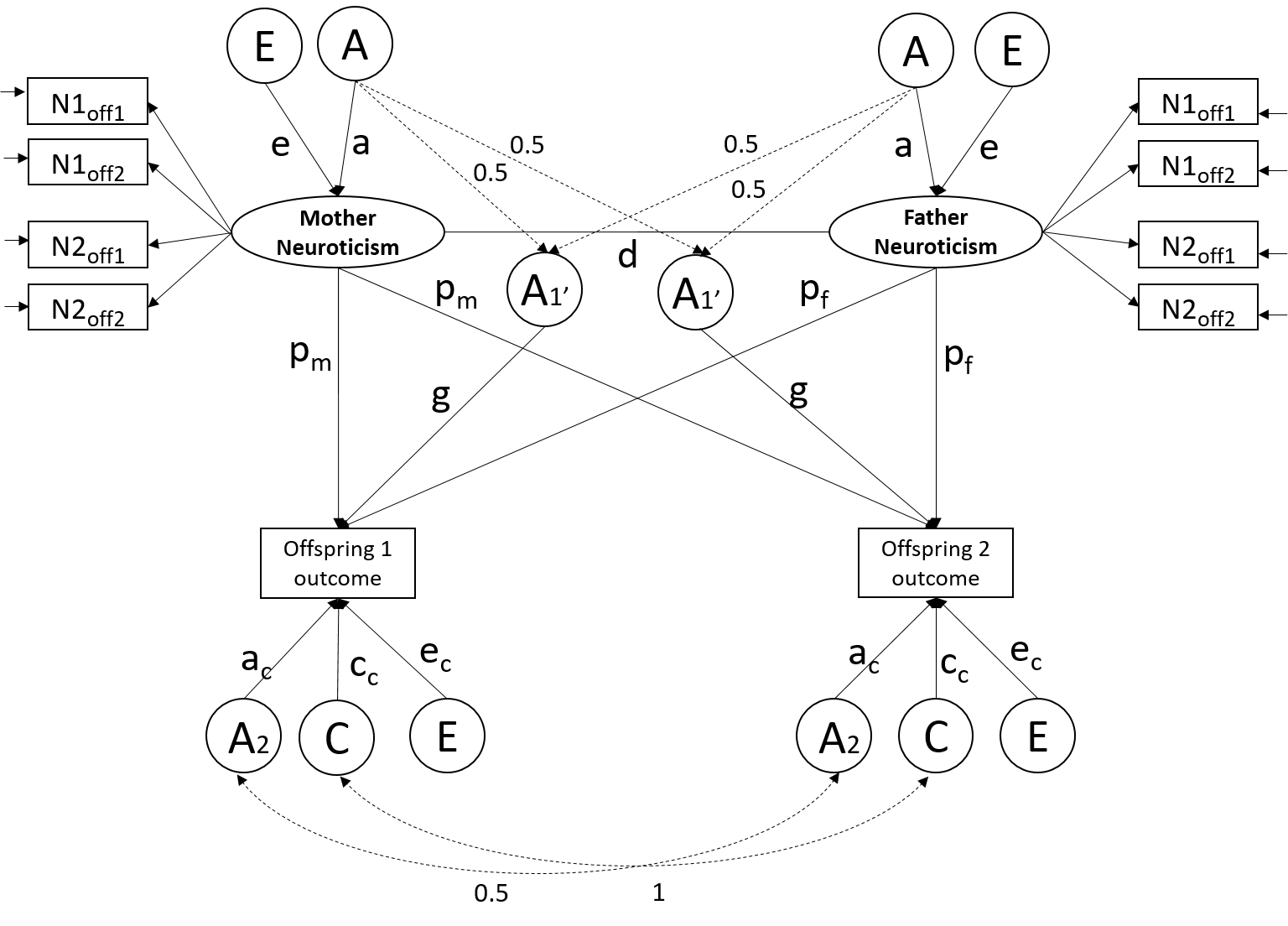


For simplicity, we show only one family unit of related family unit pairs (with siblings in the parental generation). Of main interest for our aims are the estimates of direct phenotypic transmission from parents on offspring (p_m_, p_f_), genetic transmission (g), and the assortative mating copath (d). A= Additive genetic influences, C=Common environmental influences (that make family members similar), E=Unique (non-shared) environmental influences (that make family members different, also including measurement error). N1= The IPIP measure of neuroticism, N2= The composite measure of neuroticism. All latent variables (except parental neuroticism) were modelled as having unit variance so that the square of the regression coefficient equals the variance induced by that variable. In offspring, there are two A’s, one common to parental neuroticism (A_1_’) and one specific to the offspring phenotype (A_2_).

# TableS4: Descriptive statistics

|  | Total MoBa sample | | | | |  | Pedigree sample | | | | |
| --- | --- | --- | --- | --- | --- | --- | --- | --- | --- | --- | --- |
| OFFSPRING | **N** | **mean** | **SD** | **Min** | **Max** |  | **N** | **mean** | **SD** | **Min** | **Max** |
| Neuroticism | 43367 | 2,15 | 0,67 | 1 | 5 |  | 12999 | 2,14 | 0,66 | 1 | 4,8 |
| Symptoms of anxiety | 43428 | 1,21 | 0,24 | 1 | 3 |  | 13007 | 1,2 | 0,24 | 1 | 3 |
| Symptoms of depression | 43351 | 1,14 | 0,19 | 1 | 3 |  | 12989 | 1,14 | 0,18 | 1 | 2,9 |
| Birth year | 114314 | 2005,08 | 2,21 | 1999 | 2009 |  | 29420 | 2005,41 | 1,94 | 2000 | 2009 |
| N girls (% of sample) | 54355 (48.7) |  |  |  |  |  | 14375 (48.8) |  |  |  |  |
| MATERNAL |  |  |  |  |  |  |  |  |  |  |  |
| Neuroticism (N1) | 27039 | 2,61 | 0,73 | 1 | 5 |  | 8127 | 2,58 | 0,72 | 1 | 5 |
| Symptoms of anxiety and depression (N2) | 94855 | 1,27 | 0,35 | 1 | 4 |  | 25801 | 1,25 | 0,33 | 1 | 4 |
| Self-esteem (N2) | 94966 | 1,78 | 0,49 | 1 | 4 |  | 25805 | 1,76 | 0,47 | 1 | 4 |
| Anger (N2) | 90689 | 2,11 | 0,72 | 1 | 5 |  | 25583 | 2,09 | 0,71 | 1 | 5 |
|  |  |  |  |  |  |  |  |  |  |  |  |
| PATERNAL |  |  |  |  |  |  |  |  |  |  |  |
| Neuroticism (N1) | 34919 | 2,37 | 0,64 | 1 | 5 |  | 11599 | 2,36 | 0,64 | 1 | 4,9 |
| Symptoms of anxiety and depression (N2) | 77854 | 1,15 | 0,27 | 1 | 4 |  | 26168 | 1,14 | 0,25 | 1 | 4 |
| Self-esteem (N2) | 77764 | 1,52 | 0,46 | 1 | 4 |  | 26137 | 1,51 | 0,45 | 1 | 4 |
| Anger (N2) | 78102 | 1,89 | 0,45 | 1 | 5 |  | 26212 | 1,88 | 0,44 | 1 | 4 |

# ResultsS2

All parameter estimates from the full model are presented in TableS5. The estimates of either common environmental effects in the offspring (c_c_) or childhood specific genetic influences (a_c_) have very wide confidence intervals in the models. Therefore, we ran and compared our models with and without offspring c_c_ effects. As this source of variability is defined to be orthogonal to the other sources, omitting this component should not affect any coefficient related to intergenerational transmission (direct maternal, direct paternal, and genetic transmission). This can be seen by comparing the TableS5 (with “child C”) and TableS6 (without “child C”). However, as seen in these tables, the magnitude of genetic and unique environmental effects specific to children may depend on the inclusion of “child C”. Including “child C” led to some unexpected results, for example absence of genetic effects for depressive symptoms. We interpreted this as an indication that we are not adequately powered to distinguish these two components of variance. Therefore, we retained additive genetic effects in our main models because they are apriori expected. A comparison of the fit of the two alternative models are shown in TableS7. The comparison of models with different assumptions for the intergenerational transmission turned out similar in models with and without Cc (see TableS8 for model fitting including c_c_). But it seems that we do not have the power to separate between the amount of variation in offspring traits that is explained by childhood-specific genetic and environmental influences (after accounting for intergenerational effects). In our paper, we provide results based on the models excluding the Cc.

As a sensitivity analyses, we ran all our models (with and without Cc included) using only N1 (the IPIP items) as a measure of neuroticism. Estimates from the full and the final model (with Cc) are presented in TableS9, with model fitting results in TableS10. Corresponding results without Cc are presented in TableS11 and TableS12. Generally – the intergenerational correlations were lower when including only the IPIP measure. The main conclusions from these results are, however, similar to the original models – that after adjusting for genetic confounding, there are direct maternal influences. The most striking difference is, however, the lower estimated partner correlation in the IPIP-only models. The assortative mating is estimated to be 0.02, as compared with 0.26 using the composite score. This is likely due to the different points in time for measuring the IPIP trait (fathers during pregnancy and mothers 5 years later). Due to this difference in partner similarity, the genetic transmission (that is constrained to be equal in maternal and paternal models) in the IPIP-only models is estimated to be higher in both the mother-offspring and father-offspring models.

In TableS13 and TableS14, results of models with or without an estimate of assortative mating (d) is presented.

|  | | Neuroticism | | Symptoms of depression | | Symptoms of anxiety | |
| --- | --- | --- | --- | --- | --- | --- | --- |
| Parameters | | **Full (1)** | **Final (2b)** | **Full (1)** | **Final (2b)** | **Full (1)** | **Final (3)** |
| p_m_ | Direct maternal | .32 (.21,.43) | .36 (.31,.42) | .34 (.24,.45) | .36 (.30,.41) | .25 (.14,.36) | .24 (.21,.27) |
| p_f_ | Direct paternal | -.05 (-.15,.06) | - | -.02 (-.12,.09) | - | .03 (-.07,.14) | - |
| g | Genetic transmission | .25 (.03,.46) | .16 (.09,.23) | .21 (.00,.41) | .18 (.11,.25) | -.03 (-.25,.19) | - |
| a_c_ | Child specific A | .47 (.36,.59) | .49 (.41,.58) | .00 (-.55,.55) | .00 (-.58,.58) | .22 (-.39,.84) | .22 (-1.88,2.31) |
| c_c_ | Child C | .00 (-.53,.53) | .00 (-.46,.46) | .53 (.47,.58) | .53 (.48,.57) | .36 (.15,.57) | .36 (-.30,1.02) |
| e_c_ | Child E | .81 (.76,.86) | .81 (.76,.86) | .80 (.76,.83) | .80 (.77,.83) | .84 (.77,.90) | .88 (.62,1.13) |

13. TableS5. Unstandardized Parameter estimates from the full and the final model across childhood outcomes (with Cc in the model)

|  | | Neuroticism | | Symptoms of depression | | Symptoms of anxiety | |
| --- | --- | --- | --- | --- | --- | --- | --- |
| Parameters | | **Full (1)** | **Final (2b)** | **Full (1)** | **Final (2b)** | **Full (1)** | **Final (3)** |
| p_m_ | Direct maternal | .32 (.21,.43) | .36 (.31,.42) | .35 (.25,.45) | .36 (.31,.41) | .25 (.15,.36) | .24 (.21,.27) |
| p_f_ | Direct paternal | -.05 (-.15,.06) | - | -.01 (-.11,.09) | - | .04 (-.07,.14) | - |
| g | Genetic transmission | .25 (.03,.46) | .16 (.09,.23) | .20 (.00,.40) | .18 (.11,.25) | -.04 (-.25,.17) | - |
| a_c_ | Child specific A | .47 (.36,.59) | .49 (.41,.58) | .65 (.58,.72) | .65 (.59,.71) | .52 (.43,.61) | .52 (.43,.61) |
| c_c_ | Child C | - | - | - | - | - | - |
| e_c_ | Child E | .81 (.76,.86) | .81 (.76,.86) | .70 (.65,.75) | .70 (.65,.75) | .82 (.76,.88) | .82 (.76,.88) |

14. TableS6. Unstandardized Parameter estimates from the full and the final model across childhood outcomes (without Cc in the model)

| 8 year outcome |  | Δ-2LL | Δ-df | AIC | Δ-AIC | p |
| --- | --- | --- | --- | --- | --- | --- |
| *NEUROTICISM* | with c_c_ |  |  | 45843.70 |  |  |
|  | without c_c_ | 0.00 | 1 | 45841.70 | 2 | 0.334 |
| *DEPRESSION* | with c_c_ |  |  | 45949.57 |  |  |
|  | without c_c_ | 24.75 | 1 | 45972.32 | 22.75 | <0.001 |
| *ANXIETY* | with c_c_ |  |  | 45922.15 |  |  |
|  | without c_c_ | 3.11 | 1 | 45923.26 | 1.11 | 0.078 |

15. TableS7. Model comparison: Full model with offspring environmental influences (Cc) and without Cc.

| 8 year outcome | Model | | Comparison | Δ 2LL | Δ df | AIC | Δ AIC | p |
| --- | --- | --- | --- | --- | --- | --- | --- | --- |
| *NEUROTICISM* | 1 | Full model |  |  |  | 45843.70 |  |  |
|  | 2a | No direct maternal transmission (p_m_ = 0) | 1 | 39.49 | 1 | 45881.19 | 37.49 | <0.001 |
|  | 2b | No direct paternal transmission (p_f_ = 0) | 1 | 0.70 | 1 | 45842.40 | 1.30 | 0.401 |
|  | 2c | No direct transmission (p_m_ = p_f_ = 0) | 1 | 175.88 | 2 | 46015.58 | 171.88 | <0.001 |
|  | 3 | No genetic transmission (g = 0) | 1 | 4.86 | 1 | 45846.56 | 2.86 | 0.027 |
| *DEPRESSION* | 1 | Full model |  |  |  | 45949.57 |  |  |
|  | 2a | No direct maternal transmission (p_m_ = 0) | 1 | 58.21 | 1 | 46005.78 | 56.21 | <0.001 |
|  | 2b | No direct paternal transmission (p_f_ = 0) | 1 | 0.09 | 1 | 45947.66 | 1.91 | 0.764 |
|  | 2c | No direct transmission (p_m_ = p_f_ = 0) | 1 | 164.80 | 2 | 46110.37 | 160.80 | <0.001 |
|  | 3 | No genetic transmission (g = 0) | 1 | 3.92 | 1 | 45951.48 | 1.91 | 0.048 |
| *ANXIETY* | 1 | Full model |  |  |  | 45922.15 |  |  |
|  | 2a | No direct maternal transmission (p_m_ = 0) | 1 | 19.08 | 1 | 45939.24 | 17.09 | <0.001 |
|  | 2b | No direct paternal transmission (p_f_ = 0) | 1 | 0.35 | 1 | 45920.51 | 1.64 | 0.552 |
|  | 2c | No direct transmission (p_m_ = p_f_ = 0) | 1 | 63.56 | 2 | 45981.71 | 59.56 | <0.001 |
|  | 3 | No genetic transmission (g = 0) | 1 | 0.07 | 1 | 45920.23 | 1.92 | 0.785 |

16. TableS8**. Results of the model fitting (when Cc is included in the models)** for the intergenerational transmission of parental neuroticism to offspring neuroticism, symptoms of depression, and symptoms of anxiety. -2LL: minus twice the log likelihood; AIC: Akaike Information Criterion

|  | | Neuroticism | | Symptoms of depression | | Symptoms of anxiety | |
| --- | --- | --- | --- | --- | --- | --- | --- |
| Parameters | | **Full (1)** | **Final (2b)** | **Full (1)** | **Final (2b)** | **Full (1)** | **Final (3)** |
| p_m_ | Direct maternal | .19 (.06,.32) | .24 (.19,.29) | .26 (.14,.38) | .22 (.16,.27) | .09 (-.06,.25) | .11 (.05,.16) |
| p_f_ | Direct paternal | -.05 (-.19,.08) | - | .05 (-.07,.17) | - | -.01 (-.77,.14) | - |
| g | Genetic transmission | .42 (.11,.74) | .30 (.21,.39) | .14 (-.14,.43) | .27 (.18,.36) | .15 (-.22,.51) | .12 (.04,.20) |
| a_c_ | Child specific A | .38 (.14,.61) | .44 (.32,.56) | .00 (-.59,.48) | .00 (-.48,.48) | .24 (-.33,.82) | .25 (-.34,.85) |
| c_c_ | Child C | .00 (-.38,.38) | .00 (-.39,.39) | -.54 (-.60,.-49) | -.54 (-.59,-.48) | .36 (.15,.57) | -.36 (-.58,-.13) |
| e_c_ | Child E | .80 (.74,.87) | .81 (.76,.87) | .81 (.77,.84) | .79 (.76,.83) | .87 (.79,.96) | .87 (.78,.96) |

17. TableS9**.** Unstandardized Parameter estimates (with 95% CIs) from the full and the final model across childhood outcomes (with Cc in the model) using only IPIP items to measure Neuroticism

| 8-year outcome | Model | | Comparison | Δ 2LL | Δ df | AIC | Δ AIC | p |
| --- | --- | --- | --- | --- | --- | --- | --- | --- |
| *NEUROTICISM* | 1 | Full model |  |  |  | 72961.13 |  |  |
|  | 2a | No direct maternal transmission (p_m_ = 0) | 1 | 8.96 | 1 | 72968.10 | 6.97 | 0.003 |
|  | 2b | No direct paternal transmission (p_f_ = 0) | 1 | 0.53 | 1 | 72959.67 | 1.46 | 0.465 |
|  | 2c | No direct transmission (p_m_ = p_f_ = 0) | 1 | 87.56 | 2 | 73044.70 | 83.57 | <0.001 |
|  | 3 | No genetic transmission (g = 0) | 1 | 5.91 | 1 | 72965.04 | 3.91 | 0.015 |
| *DEPRESSION* | 1 | Full model |  |  |  | 73133.21 |  |  |
|  | 2a | No direct maternal transmission (p_m_ = 0) | 1 | 24.34 | 1 | 73155.55 | 22.34 | <0.001 |
|  | 2b | No direct paternal transmission (p_f_ = 0) | 1 | 0.77 | 1 | 73131.99 | 1.22 | 0.379 |
|  | 2c | No direct transmission (p_m_ = p_f_ = 0) | 1 | 66.07 | 2 | 73195.29 | 62.08 | <0.001 |
|  | 3 | No genetic transmission (g = 0) | 1 | 0.95 | 1 | 73132.17 | 1.04 | 0.329 |
| *ANXIETY* | 1 | Full model |  |  |  | 72982.61 |  |  |
|  | 2a | No direct maternal transmission (p_m_ = 0) | 1 | 1.39 | 1 | 72981.99 | 0.62 | 0.239 |
|  | 2b | No direct paternal transmission (p_f_ = 0) | 1 | 0.02 | 1 | 72980.63 | 1.98 | 0.886 |
|  | 2c | No direct transmission (p_m_ = p_f_ = 0) | 1 | 16.72 | 2 | 72995.32 | 12.71 | <0.001 |
|  | 3 | No genetic transmission (g = 0) | 1 | 0.60 | 1 | 72981.21 | 1.4 | 0.439 |

18. TableS10. Results of the model fitting (when Cc is included in the models) for the intergenerational transmission of parental neuroticism (including only IPIP items to measure Neuroticism) to offspring neuroticism, symptoms of depression, and symptoms of anxiety. -2LL: minus twice the log likelihood; AIC: Akaike Information Criterion

|  | | Neuroticism | | Symptoms of depression | | Symptoms of anxiety | |
| --- | --- | --- | --- | --- | --- | --- | --- |
| Parameters | | **Full (1)** | **Final (2b)** | **Full (1)** | **Final (2b)/(3)** | **Full (1)** | **Final (2b)** |
| p_m_ | Direct maternal | .19 (.05,.33) | .24 (.19,.29) | .28 (.16,.41) | .22 (.17,.27)/.32 (.29,.36) | .10 (-.07,.26) | .11 (.06,.16) |
| p_f_ | Direct paternal | -.05 (-.19,.09) | - | .07 (-.06,.20) | - /.11 (.07,.11) | -.01 (-.18,.16) | - |
| g | Genetic transmission | .42 (.10,.75) | .30 (.21,.39) | .10 (-.19,.40) | .26 (.17,.35)/ - | .14 (-.26,.54) | .12 (.04,.20) |
| a_c_ | Child specific A | .38 (.14,.62) | .44 (.33,.55) | .67 (.61,.74) | .65 (.58,.72)/.68 (.62,.74) | .53 (.41,.64) | .53 (.43,.63) |
| c_c_ | Child C | - | - | - | - | - | - |
| e_c_ | Child E | .80 (.74,.87) | .81 (.76,.87) | .71 (.65,.76) | .70 (.65,.76)/.71 (.65,.76) | .82 (.75,.88) | .82 (.76,.88) |

19. TableS11**.** Unstandardized Parameter estimates (with 95% CIs) from the full and the final model across childhood outcomes (without Cc in the model) using only IPIP items to measure Neuroticism

| 8-year outcome | Model | | Comparison | Δ 2LL | Δ df | AIC | Δ AIC | p |
| --- | --- | --- | --- | --- | --- | --- | --- | --- |
| *NEUROTICISM* | 1 | Full model |  |  |  | 72959.13 |  |  |
|  | 2a | No direct maternal transmission (p_m_ = 0) | 1 | 8.96 | 1 | 72966.10 | 6.97 | <0.001 |
|  | 2b | No direct paternal transmission (p_f_ = 0) | 1 | 0.53 | 1 | 72957.67 | 1.46 | 0.465 |
|  | 2c | No direct transmission (p_m_ = p_f_ = 0) | 1 | 87.56 | 2 | 73042.70 | 83.57 | <0.001 |
|  | 3 | No genetic transmission (g = 0) | 1 | 5.91 | 1 | 72963.04 | 3.91 | 0.015 |
| *DEPRESSION* | 1 | Full model |  |  |  | 73154.92 |  |  |
|  | 2a | No direct maternal transmission (p_m_ = 0) | 1 | 22.32 | 1 | 73175.24 | 20.32 | <0.001 |
|  | 2b | No direct paternal transmission (p_f_ = 0) | 1 | 1.24 | 1 | 73154.15 | 0.77 | 0.662 |
|  | 2c | No direct transmission (p_m_ = p_f_ = 0) | 1 | 68.92 | 2 | 73219.83 | 64.91 | <0.001 |
|  | 3 | No genetic transmission (g = 0) | 1 | 0.48 | 1 | 73153.39 | 1.53 | 0.490 |
| *ANXIETY* | 1 | Full model |  |  |  | 72983.09 |  |  |
|  | 2a | No direct maternal transmission (p_m_ = 0) | 1 | 1.30 | 1 | 72982.40 | 17.09 | 0.254 |
|  | 2b | No direct paternal transmission (p_f_ = 0) | 1 | 0.02 | 1 | 72981.11 | 1.64 | 0.902 |
|  | 2c | No direct transmission (p_m_ = p_f_ = 0) | 1 | 16.80 | 2 | 72995.89 | 59.56 | <0.001 |
|  | 3 | No genetic transmission (g = 0) | 1 | 0.52 | 1 | 72981.61 | 1.92 | 0.472 |

20. TableS12. **Results of the model fitting (when Cc is not included in the models)** for the intergenerational transmission of parental neuroticism (including only IPIP items to measure neuroticism) to offspring neuroticism, symptoms of depression, and symptoms of anxiety. -2LL: minus twice the log likelihood; AIC: Akaike Information Criterion; Best fitting models are marked in bold.

| 8 year outcome |  | Δ-2LL | Δ-df | AIC | Δ-AIC | p |
| --- | --- | --- | --- | --- | --- | --- |
| *NEUROTICISM* | Model 1 |  |  | 45843.70 |  |  |
|  | Model 1 with d=0 | 627.80 | 1 | 46469.50 | 625,80 | <0.001 |
| *DEPRESSION* | Model 1 |  |  | 45949.57 |  |  |
|  | Model 1 with d=0 | 661.45 | 1 | 46609.01 | 659.44 | <0.001 |
| *ANXIETY* | Model 1 |  |  | 45922.15 |  |  |
|  | Model 1 with d=0 | 636.82 | 1 | 46556.97 | 634.82 | <0.001 |

21. TableS13**. Model comparison of models including or excluding assortative mating (d=0)**

|  | Neuroticism | Symptoms of depression | Symptoms of anxiety |
| --- | --- | --- | --- |
| p_m_ | .28 (.16,.40) | .34 (.22,.46) | .25 (.12,.39) |
| p_f_ | -.05 (-.17,.06) | .01 (-.10,.13) | .06 (-.08,.20) |
| g | .31 (.09,.54) | .20 (-.03,.43) | -.06 (-.33,.22) |

22. TableS14. Unstandardized Parameter estimates (SE) from the full model across childhood outcomes, if d = 0.


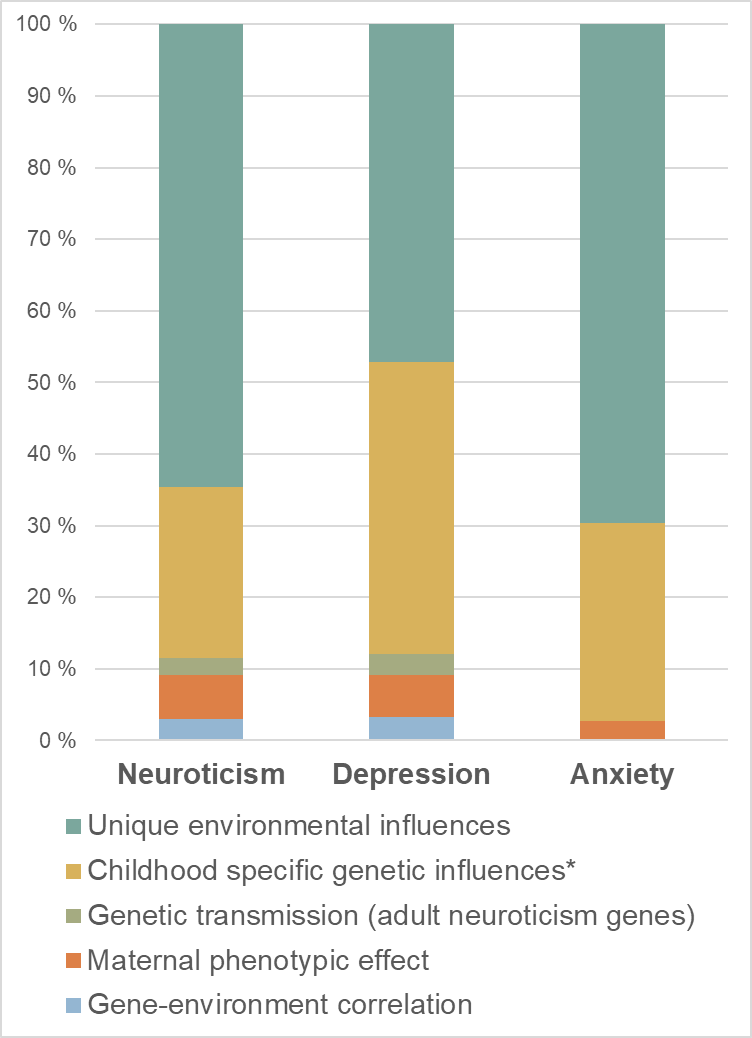


23. FigS5. Percentage of variance explained in 8-year-old children’s scores on neuroticism, symptoms of anxiety, and symptoms of depression by intergenerational and child-specific influences.

# **References**

D'Onofrio, B. M., Turkheimer, E. N., Eaves, L. J., Corey, L. A., Berg, K., Solaas, M. H., & Emery, R. E. (2003). The role of the children of twins design in elucidating causal relations between parent characteristics and child outcomes. *Journal of Child Psychology and Psychiatry, 44*(8), 1130-1144.

Jorgensen, T., Pornprasertmanit, S., Schoemann, A., Rosseel, Y., Miller, P., Quick, C., & Mansolf, M. (2018). semTools: Useful tools for structural equation modeling (R package version 0.5-1)[Computer software]. In.

McAdams, T. A., Neiderhiser, J. M., Rijsdijk, F. V., Narusyte, J., Lichtenstein, P., & Eley, T. C. (2014). Accounting for genetic and environmental confounds in associations between parent and child characteristics: A systematic review of children-of-twins studies. *Psychological bulletin, 140*(4), 1138-1173. Retrieved from <http://psycnet.apa.org/journals/bul/140/4/1138.pdf>

Muthén, L. (2010). Muthén: BO Mplus User’s Guide. *Los Angeles, CA: Muthén and Muthén*.

Neale, M. C., & Maes, H. H. M. (2004). *Methodology for genetic studies of twins and families*. Dordrecht, The Netherlands: Kluwer Academic Publishers B.V.

Silberg, J. L., Maes, H., & Eaves, L. J. (2010). Genetic and environmental influences on the transmission of parental depression to children’s depression and conduct disturbance: an extended Children of Twins study. *Journal of Child Psychology and Psychiatry, 51*(6), 734-744.

Ystrom, E., Barker, M., & Vollrath, M. E. (2012). Impact of mothers' negative affectivity, parental locus of control and child‐feeding practices on dietary patterns of 3‐year‐old children: The MoBa Cohort Study. *Maternal & Child Nutrition, 8*(1), 103-114.
